# HER3 in cancer: from the bench to the bedside

**DOI:** 10.1186/s13046-022-02515-x

**Published:** 2022-10-21

**Authors:** Lucía Gandullo-Sánchez, Alberto Ocaña, Atanasio Pandiella

**Affiliations:** 1 Instituto de Biología Molecular y Celular del Cáncer, CSIC, IBSAL and CIBERONC, Campus Miguel de Unamuno, 37007 Salamanca, Spain; 2 Hospital Clínico San Carlos and CIBERONC, 28040 Madrid, Spain

**Keywords:** HER3, Cancer therapy, Receptor tyrosine kinases

## Abstract

The HER3 protein, that belongs to the ErbB/HER receptor tyrosine kinase (RTK) family, is expressed in several types of tumors. That fact, together with the role of HER3 in promoting cell proliferation, implicate that targeting HER3 may have therapeutic relevance. Furthermore, expression and activation of HER3 has been linked to resistance to drugs that target other HER receptors such as agents that act on EGFR or HER2. In addition, HER3 has been associated to resistance to some chemotherapeutic drugs. Because of those circumstances, efforts to develop and test agents targeting HER3 have been carried out. Two types of agents targeting HER3 have been developed. The most abundant are antibodies or engineered antibody derivatives that specifically recognize the extracellular region of HER3. In addition, the use of aptamers specifically interacting with HER3, vaccines or HER3-targeting siRNAs have also been developed. Here we discuss the state of the art of the preclinical and clinical development of drugs aimed at targeting HER3 with therapeutic purposes.

## Background

The ErbB/HER receptor tyrosine kinases (RTK) play critical roles in animal development, and their altered function may contribute to the pathophysiological development of certain types of tumors [1, 2]. In mammals, four ErbB/HER receptors have been described: the epidermal growth factor receptor (EGFR/HER1), HER2/ErbB2/neu, HER3/ErbB3, and HER4/ErbB4 [3]. These receptors are physiologically expressed in epithelial, mesenchymal, cardiac, and neuronal tissues.

Overexpression of HER2 in a subgroup of breast tumors [4], together with preclinical evidence of an oncogenic role of this transmembrane protein [5], encouraged the development of agents targeting such receptor. These efforts led to the arrival to the clinic of agents, such as the humanized monoclonal antibody trastuzumab, that by targeting HER2 offered clinical benefit [6]. The clinical success of this strategy led later to the development of agents that targeted the cognate receptor EGFR [7]. The clinical development of agents targeting other ErbB receptors is on the rise due to their suspected role in tumorigenesis or therapy resistance. Thus, expression or overexpression of HER3 has been reported in many cancers, such as breast, ovarian, lung, colorectal, melanoma, head and neck, cervical and prostate cancers [8–12]. Moreover, several studies have pointed to HER3 as a major determinant in resistance to certain therapies, some of them targeting other ErbB receptors [13]. The expression of HER3 in tumors opens the possibility of its targeting with therapeutic purposes. In this review we will discuss the biological bases behind the design of anti-HER3 therapies as well as the clinical status of agents that target this receptor.

## HER3: structure, activation, and physiological role

**Fig. 1:**
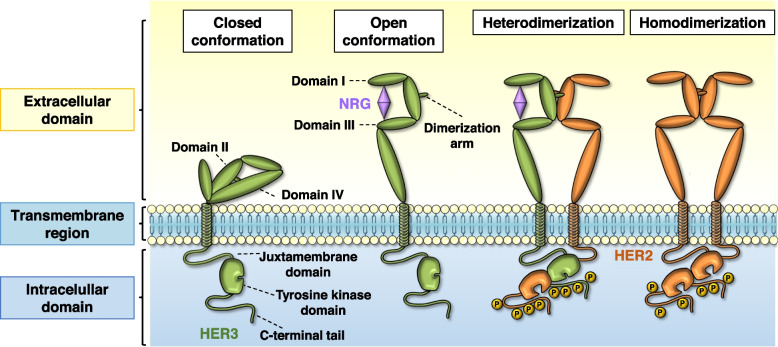
Schematic representation of the structural changes and activation of ErbB/HER receptors. This family is compromised by four members. Each member is composed of an extracellular region, a transmembrane region, and an intracellular region. The extracellular region, in turn, is composed of four subdomains (I-IV). The intracellular region contains the juxtamembrane domain, the tyrosine kinase domain, and the C-terminal tail with phosphorylatable residues. When the ligand binds to subdomains I and III, a conformational change is induced in the extracellular domain, leaving the dimerization arm exposed. Thanks to this, the receptor can dimerize with another member of the family in open conformation (heterodimerization) or another identical receptor (homodimerization)

HER3, identified by Kraus et al. [14], is encoded by the *ERBB3* gene and maps to the human chromosome 12q13. HER3 is widely expressed in human adult tissues, including cells of the gastrointestinal, urinary, respiratory, reproductive tracts, skin, endocrine and nervous systems [15]. HER3 consists of a large extracellular domain (ECD), a single hydrophobic transmembrane segment, and an intracellular domain that includes a juxtamembrane region, a tyrosine kinase segment, and a tyrosine-rich carboxyterminal tail (Fig. 1) [16, 17]. The extracellular domain consists of four subdomains, referred to as subdomains I-IV [3].

Physiological activation of HER3 can be triggered by its interaction with the neuregulins (NRGs), a group of polypeptides that belong to the EGF family of ligands [18, 19]. In the absence of ligand, a direct intramolecular interaction between subdomains II and IV keeps HER3 in an inactive (closed or tethered) conformation [20]. Ligand binding to subdomains I and III provokes a structural change of the extracellular region of the receptor, which acquires an open conformation [21]. Such conformational change results in exposure of the dimerization arm, located in subdomain II. The dimerization arm then allows intermolecular interaction with another ErbB RTK monomer to form dimeric complexes (Fig. 1). Ligand binding also results in changes in the intracellular disposition of the ErbB receptors. Thus, the two kinase domains interact in an asymmetric “head to tail” conformation in which one kinase allosterically activates the other [22, 23].

A debated aspect of HER3 relates to its kinase activity. Initially, it was reported that HER3 lacked kinase activity due to the absence of critical residues necessary for that activity. Later, several reports indicated that HER3 had in fact some tyrosine kinase activity [24]. Although HER3 homodimers have been reported [25–28], HER3 preferentially dimerizes with other ErbB family members, especially HER2. Indeed, ligand-independent HER2-HER3 heterodimers have also been reported in HER2-amplified (HER2 +) cells [29]. However, such interactions are expected to be weaker and shorter lasting, if compared to ligand-induced dimerization. In fact, studies on the interaction of HER3 and HER2 in breast cancer cells showed that both receptors could only form stable dimers when the HER3 ligand NRG was present [30]. That circumstance opens the relevant question as to how HER3 is constitutively tyrosine phosphorylated in HER2 overexpressing cells. Perhaps, that could be explained by a short but frequent kiss-and-run interaction between HER2 and HER3.

## HER3 expression in tumors and clinical outcomes

HER3 expression or overexpression has been described in multiple types of tumors, including breast [31], ovarian [32, 33], lung [11], colon [34], pancreatic [10], melanoma [35], gastric [9, 36], head and neck [37] and prostate cancers [12]. Analysis of the TCGA dataset using the cBioportal online tool (accessed June 2022) shows that melanomas represent the tumor type with the highest HER3 expression at the mRNA level, followed by cholangiocarcinomas and invasive breast tumors. Melanoma metastases commonly have greater HER3 expression than primary tumors [35]. HER3 overexpression has also been found in pilocytic astrocytoma, a childhood glioma, [38] and in rhabdomyosarcoma, a pediatric sarcoma [39].

Ocaña et al. performed a meta-analysis evaluating the association of HER3 expression and patient outcome in solid tumors using published information [8]. It was observed that HER3 was overexpressed in 42% of the tumors and in some of them, including melanoma, cervical, or ovarian cancers, HER3 was highly expressed in more than 50% of the cases. In addition, HER3 was associated with worse overall survival in several tumors, especially in HER2-overexpressing cancers. HER3 is overexpressed in human papillomavirus positive (HPV +) models of human tumors and is a prognostic factor for poor outcome in pharyngeal cancer [40]. HER3 is also overexpressed in some prostate cancers [41, 42] and is associated with poor prognosis [12]. Additional studies reported that HER3 overexpression is related with poor prognosis in non-small cell lung cancers (NSCLC) and decreased survival in early-stages [11, 43, 44].

Overexpression of HER3 is often associated with overexpression of HER2 and/or EGFR, playing an important role as co-receptor in HER2 + breast cancer and in a subset of EGFR-positive lung tumors [45–48]. Furthermore, breast cancers often show co-expression and positive correlation between HER2 and HER3 [49, 50]. This co-expression leads to decreased patient survival [51]. In addition, HER3 is significantly expressed in estrogen receptor positive (ER +) or luminal breast cancer, being essential for cell survival in the luminal but not basal breast epithelium [52, 53].

Finally, little data is reported regarding the presence of oncogenic mutations of *ERBB3.* These mutations have been mostly reported in gastric and colon adenocarcinomas, and less frequently in NSCLC. Mutant *ERBB3* oncogenic forms appear to be ligand-independent and require HER2 [54]. Currently, *ERBB3* mutations are on study due to potential therapeutic implications [55–58].

## Biological role of HER3 in therapeutic resistance

HER3 has been implicated in resistance to therapies targeting other HER receptors as well as in resistance to chemotherapies.

When a certain ErbB receptor is blocked, other RTKs may compensate the signaling lost by the blocked receptor. For example, when EGFR is targeted with small molecule tyrosine kinase inhibitors (TKIs) and resistance develops, the signaling blockade can be overcome by an increase in HER3 expression [59] or amplification of another receptor kinase like MET [46]. Resistance to the anti-EGFR antibody cetuximab in lung cancer is also associated with deregulation of EGFR internalization/degradation and may be associated to activation of HER3 [60]. Also, HER3 signaling has been linked to resistance to TKIs targeting the EGFR in head and neck squamous cell carcinoma (HNSCC) [61]. Huang et al. found that the heterotrimeric HER2-HER3/IGF1R leads to trastuzumab resistance triggering PI3K/AKT and Src kinase signaling [62]. Upregulation of HER3 expression or signaling have also been associated with resistance to lapatinib or trastuzumab in HER2 + breast cancer [63–67].

The expression of the HER3 ligands has been reported to facilitate activation of HER3 leading to resistance to agents targeting other HER receptors. Thus, increased expression and activation of HER3 accompanied by expression of NRG have been reported in HER2 + breast cancer cells resistant to the antibody–drug conjugate (ADC) trastuzumab-emtansine (T-DM1) [68]. This increase in the expression/signaling of HER3 has also been associated with resistance to the insulin-like growth factor 1 receptor (IGF1R) inhibitors in hepatocarcinoma [69]. In this line, high expression of NRG has been reported to be a possible mechanism of resistance to cetuximab in colorectal cancer [70]. Interestingly, in a subset of ovarian cancers, autochtonous production of NRG has been discovered to stimulate proliferation via an autocrine loop involving NRG and HER3 [71].

As mentioned above, besides its role in resistance to targeted therapies, HER3 may also play a role in resistance to chemotherapy. In HER2 + breast cancer, elevated HER3 expression results in resistance to paclitaxel via upregulation of survivin [72]. Moreover, co-expression of HER2 and HER3 in breast cancer cell lines was associated with resistance to a broad-spectrum of chemotherapeutic agents, likely through up-regulation of PI3K/AKT signaling [73]. HER3 signaling and expression may also play a role in the development of chemoresistance in ovarian cancer [74, 75]. In prostate cancer, HER3/PI3K/AKT signaling has been implicated in the development of hormone resistance and progression to docetaxel resistance [76]. HER3 has also been reported to play a significant role in anti-estrogen (fulvestrant, tamoxifen) resistance in ER + breast cancer [77–80]. In addition, upregulation of HER3 expression has been reported to be related to resistance to RAF and MEK inhibitors in melanoma and thyroid carcinomas [81, 82].

## Current anti-HER3 therapies

**Fig. 2:**
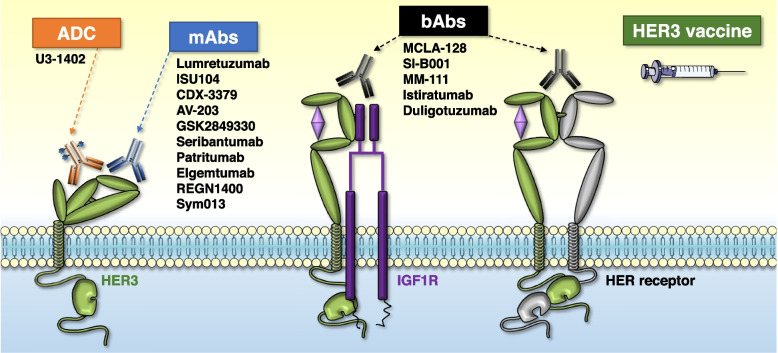
Therapies against HER3 under clinical development. Monoclonals antibodies (mAbs), bispecific antibodies (bAbs), antibody–drug conjugate (ADC) and other therapies such as HER3 vaccine

**Fig. 3:**
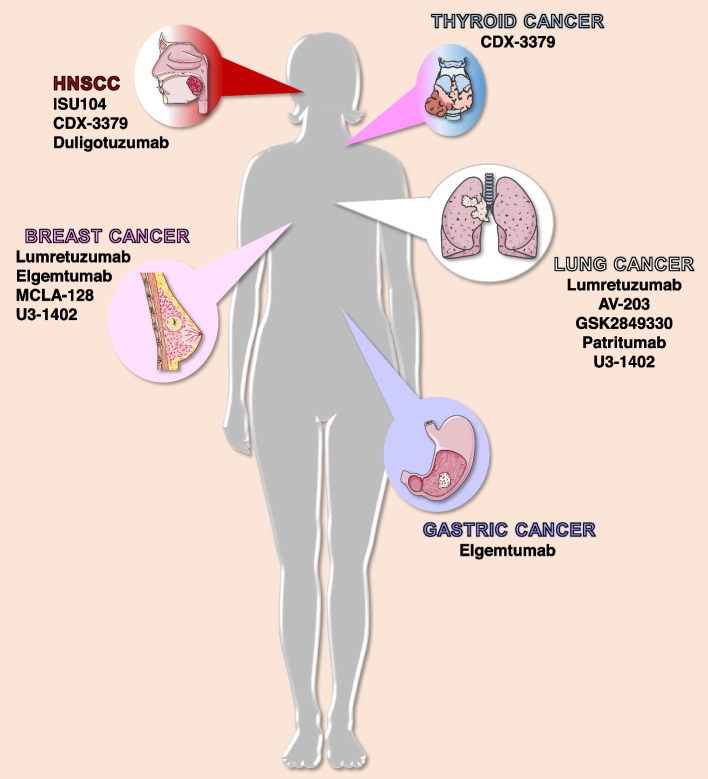
Cancers in which have been reported clinical efficacy of anti-HER3 drugs under clinical development

In the following section we will describe current strategies to target HER3, which are essentially based on the use of antibodies that recognize the extracellular region of HER3. Figure 2 shows a schematic representation of the therapies described below and Fig. 3 the tumors in which have been reported promising clinical activity.

### Monoclonal antibodies

#### Under clinical development

All clinical trials of monoclonal antibodies (mAbs) in clinical evaluation are summarized in Table 1.

**Table 1:** Monoclonal antibodies against HER3 under clinical development

**Study population**	**Clinical Trial, phase**	**Adverse events**	**Status, conclusion (references)**
**Lumretuzumab (RG7116, RO5479599, GE-huMab-HER3)**			
Advanced or metastatic NSCLC	NCT02204345, phase I + II	Gastrointestinal, hematological and nervous system toxicities, but generally mild and manageable	Terminated. No results posted Efficacy of lumretuzumab + carboplatin + paclitaxel is like chemotherapy alone [83]
Metastatic BC expressing HER3 and HER2	NCT01918254, phase Ib	Diarrhea and hypokalemia	Completed. No results posted Lumretuzumab + pertuzumab + paclitaxel was related with a serious incidence of diarrhea that cannot warrant further clinical development [84]
Metastatic and/or locally advanced malignant HER3 + solid tumors of epithelial cell origin	NCT01482377, phase I	Gastrointestinal and skin toxicities	Completed. No results posted Moderate clinical activity was observed with toxicity manageable [85, 86]
**ISU104**			
Advanced solid tumors Dose escalation study (PART I) Dose-expansion study (PART II)	NCT03552406, phase I	PART I: Oral mucositis, pruritus, diarrhea and fatigue PART II: anorexia, mucositis oral and diarrhea in monotherapy and diarrhea and acneiform rash in combination with cetuximab	Active, not recruiting PART I: ISU104 was well tolerated up to 20 mg/kg/day without DLT and showed disease control rate of 60% [87] PART II: ISU104 monotherapy or with cetuximab was safe with promising clinical outcomes in recurrent or metastatic HNSCC treated with the combination [88]
**CDX-3379 (KTN3379)**			
Advanced cancer	NCT02014909, phase I	Diarrhea, fatigue, nausea and rash	Completed. No results posted CDX-3379 can be combined in safety with cetuximab, erlotinib, vemurafenib or trastuzumab at 15 to 20 mg/kg [89]
HNSCC	NCT02473731, phase I	Diarrhea, fatigue and acneiform dermatitis, but mild or moderate	Completed. No results posted CDX-3379 was well tolerated and associated with tumor regression [90]
Advanced Stage NRAS mutant and BRAF/NRAS wildtype melanoma	NCT03580382, phase I + II		Terminated (Per regulatory coordinator, the sponsor is no longer supporting the study). Study results available online
Advanced HNSCC	NCT03254927, phase II		Completed. Resulted submitted CDX-3379 in combination with cetuximab is well tolerated with signs of antitumor activity [91]
Thyroid cancer	NCT02456701, phase I		Completed. No results posted Vemurafenib + CDX-3379 is safe and enhance efficacy for RAI uptake [92]
**AV-203 (CAN017)**			
Metastatic or advanced solid tumors	NCT01603979, phase I		Completed. No results posted AV-203 was well tolerated. RP2D is 20 mg/kg IV every 2 weeks. The PR in a patient with squamous NSCLC guarantees future testing of AV-203 in this indication [93]
**GSK2849330**			
Advanced HER3 + solid tumors	NCT01966445, phase I	Drug tolerated with no major issues	Completed. Study results available online GSK2849330 has durable response in an exceptional responder with an advanced CD74–NRG1-rearranged IMA [94]
Advanced HER3 + solid tumors	NCT02345174, phase I	Decreased appetite and diarrhea	Completed. No results posted Immuno-positron emission tomography reveals good tumor uptake in all evaluable patients. Despite the restricted number of patients, an exploratory ID_50_ of 2 mg/kg and ID_90_ of 18 mg/kg have been reported [95]
**Seribantumab (MM-121, SAR256212)**			
Advanced NSCLC	NCT00994123, phase I + II	Diarrhea, rash, decreased appetite, fatigue and nausea	Completed. Study results available online Phase I: No maximum tolerated dose was determined and the AE profile was similar between comparative treatment Phase II: there was no significant difference in PFS between monotherapy or combination therapy. However, retrospective analyses suggest that detectable NRG mRNA levels identified patients who may benefit from MM-121 [96]
NSCLC expressing NRG	NCT02387216, phase II	Diarrhea, fatigue and neutropenia in the combination treatment	Terminated (Based on the preliminary results seen during interim analysis, which were confirmed in the final analysis, the Sponsor terminated the study) Seribantumab does not improve PFS when added to docetaxel [97]
CRC, HNSCC, NSCLC, TNBC and other tumors with EGFR dependence	NCT01451632, phase I	Part 1: fatigue, dermatitis acneiform, hypomagnesemia, diarrhea, decreased appetite and hypokalemia Part 2: diarrhea, hypokalemia, nausea, fatigue, hypomagnesemia, decreased appetite, dermatitis acneiform, mucosal inflammation, dehydration and weight decrease	Completed. Study results available online Unlike doublet treatment, seribantumab + cetuximab + irinotecan was difficult to tolerate. However, MM121 + cetuximab with and without irinotecan had no activity in the vast majority patients with prior exposure to EGFR directed therapy [98, 99]
Advanced gynecologic and breast cancers	NCT01209195, phase I		Completed. Study results available online
ER + , HER2- BC and TNBC	NCT01421472, phase II		Completed. Study results available online
Platinum resistant or refractory recurrent/advanced ovarian cancers	NCT01447706, phase II	Diarrhea, vomiting, stomatitis and mucosal inflammation	Completed. Study results available online MM-121 + paclitaxel was no more effective than paclitaxel alone in prolonging nor OS neither PFS [100]. Exploratory analyses suggest that patients with detectable NRG and low HER2 might benefit from this combination [101]
Locally advanced or metastatic ER + and/or PR + and HER2- BC	NCT01151046, phase II	Diarrhea, nausea, fatigue and arthralgia	Completed. Study results available online The addition of MM-121 to exemestane did not significantly prolong PFS in the unselected population [102]
CRC, NSCLC and HNSCC	NCT02538627, phase I		Terminated (Sponsor decision). No results posted
Advanced solid tumors	NCT00734305, phase I		Completed. Study results available online
Advanced solid tumors	NCT01447225, phase I	Diarrhea, nausea, fatigue, anemia, vomiting, hypokalemia, decreased appetite, thrombocytopenia, peripheral edema, neutropenia and constipation	Completed. Study results available online MM-121 can be administrated with gemcitabine, pemetrexed, cabazitaxel and carboplatin [103]
Postmenopausal women with metastatic BC	NCT03241810, phase II		Terminated (Merrimack Inc. terminated the trial early due to business decision). Study results available online
Locally advanced or metastatic solid tumors	NCT01436565, phase I		Completed. No results posted
NRG1 gene fusion positive advanced solid tumors	NCT04383210, phase II		Recruiting
An NRG1 fusion positive metastatic pancreatic cancer patient	NCT04790695, phase II		Completed. No results posted
**Patritumab (AMG-888, U3-1287)**			
Advanced, refractory solid tumors	NCT01957280, phase I	The most frequently reported treatment-related AEs were gastrointestinal	Completed. No results posted Patritumab produced by a new manufacturing process was well tolerated with no anti–patritumab neutralizing antibodies formation and with normal bioavailability [104]
EGFR wild-type subjects with locally advanced or metastatic NSCLC who have progressed on at least one prior systemic therapy	NCT02134015, phase III	In placebo + erlotinib the most frequent AEs were rash, diarrhea and fatigue, in patritumab + erlotinib were diarrhea, rash and decreased appetite	Terminated (Pre-defined criteria for continuation were not reached). Study results available online Patritumab + erlotinib apparently do not get better results of placebo + erlotinib
Recurrent or metastatic HNSCC	NCT02633800, phase II	Rash, anemia, neutropenia, hypomagnesemia and nausea	Terminated (Trial was terminated by sponsor due to lack of efficacy). Study results available online Patritumab + cetuximab + platinum was safe but not more efficacious than cetuximab + platinum [105]
EGFR treatment naïve subjects with advanced NSCLC who have progressed on at least one prior chemotherapy	NCT01211483, phase I + II	AE grade > 3 included diarrhea and rash	Completed. Study results available online Patritumab improved PFS in the NRG high, but not in the ITT population [106]
Recurrent or metastatic HNSCC	NCT02350712, phase I	Skin and subcutaneous tissue disorders	Completed. No results posted Patritumab (18 mg/kg loading dose, 9 mg/kg maintenance dose) with cetuximab and platinum therapy was tolerated and active in HNSCC [107]
Advanced solid tumors	NCT01479023, phase I	Diarrhea, dizziness, fatigue, headache, hypertension and weight loss	Terminated (treatment was not working). No results posted This study confirmed that the administration of [64Cu]DOTA-patritumab and unlabeled patritumab is safe and well-tolerated [108]
Newly diagnosed HER2 + metastatic BC	NCT01512199, phase I + II		Terminated (Improved, different standard of care caused business decision to terminate). No results posted
Advanced solid tumors	NCT00730470, phase I	Fatigue, diarrhea, nausea, decreased appetite and dysgeusia	Completed. No results posted Patritumab treatment was well tolerated and was observed some evidence of disease stabilization [109]
**Elgemtumab (LJM716)**			
Platinum-pretreated recurrent/metastatic HNSCC	NCT02143622, phase I + II		Withdrawn
Advanced HER2 + BC or gastric cancer	NCT01602406, phase I	Diarrhea, nausea, fatigue and chills	Completed. No results posted As of October 4, 2013, LJM716 demonstrated clinical activity in combination with trastuzumab in trastuzumab-resistant patients. The safety profile of the combination was acceptable [110]
Metastatic HER2 + BC	NCT02167854, phase I	Diarrhea, hyperglycemia, hypokalemia, mucositis and transaminitis	Completed. No results posted The combination treatment of LJM716, BYL719 and trastuzumab has antitumor activity in these pre-treated HER2 + metastatic BC with *PIK3CA* mutations [111]
Patients with previously treated ESCC	NCT01822613, phase I + II		Completed. No results posted
HER2 + BC, HER2 + gastric cancer, HNSCC and ESCC	NCT01598077, phase I	Diarrhea, decreased appetite, pyrexia, fatigue, nausea, infusion-related reactions, vomiting, constipation and dyspnea and anemia and hypomagnesemia	Completed. No results posted LJM716 was well tolerated, with a manageable safety profile [112]
Japanese patients with advanced solid tumors	NCT01911936, phase I	Diarrhea, stomatitis, fatigue, pyrexia and paronychia	Completed. No results posted LJM716 was well tolerated and a degree of tumor shrinkage was reported [113]
**REGN1400**			
Patients with advanced NSCLC, CRC or HNSCC who progressed on prior erlotinib or cetuximab	NCT01727869, phase I	Rash, diarrhea, nausea and hypomagnesemia	Completed. No results posted REGN1400 as monotherapy or combined with erlotinib or cetuximab was generally tolerated [114]
**Sym013**			
Patients with advanced epithelial malignancies	NCT02906670, phase I + II		Terminated (Business reasons). Study results available online

##### Lumretuzumab (RG7116, RO5479599, GE-huMab-HER3)

Lumretuzumab is a humanized glycoengineered IgG1 directed to subdomain I of the HER3-extracellular domain [115]. The antibody prevents NRG binding and therefore receptor heterodimerization and activation. It also induces HER3 downregulation. In various tumor xenograft models, lumretuzumab alone or in combination with other anti-HER therapies, caused substantial tumor growth inhibition, including some complete remissions. Lumretuzumab binds to human FcγRIIIa on immune effector cells with more affinity than standard non-glycoengineered antibodies, provoking enhanced antibody-mediated cell-dependent cytotoxicity (ADCC). In xenograft models of ER + /HER3 + /HER2-low human breast cancers, a lumretuzumab and pertuzumab combination was potent and induced long-lasting tumor regression [116]. Indeed, an increase in efficacy was observed if fulvestrant was added. A patient with ER + /HER3 + /HER2-low breast cancer had a prolonged clinical response when she was treated with lumretuzumab + pertuzumab + paclitaxel. Recently, it has been reported that two patients benefited from lumretuzumab plus erlotinib treatment in lung cancer [85].

##### ISU104

ISU104 is a fully human anti-HER3 antibody that binds to subdomain III and is in early clinical development [87, 88]. This antibody downregulates HER3, inhibits NRG binding, blocks dimerization with other HER partners and inactivates the downstream signaling from HER3. In vivo, ISU104 showed more than 70% tumor growth inhibition in HNSCC, NSCLC, colon, pancreatic, breast and skin xenograft cancer models [117, 118]. ISU104 has also showed anti-tumor effects in acquired cetuximab-resistant xenografts either alone or in combination with cetuximab [119]. Recently, Hong et al. have reported anti-tumor efficacy of ISU104 in models with high NRG1 expression or harboring genetic alterations such as *NRG1*-fusion or oncogenic *ERBB3* mutations [120].

##### CDX-3379 (KTN3379)

CDX-3379 is a human monoclonal antibody (IgG1λ) that binds with very high affinity to a unique epitope in the boundary between domains II and III and locks HER3 in its inactive state [121, 122]. For this reason, this antibody inhibited both ligand dependent and ligand independent HER3 activation. Its Fc region contains 3 amino acid substitutions, that are referred to as YTE, which increase IgG affinity for human FcRn [123]. CDX-3379 has shown its efficacy in NRG-driven tumors, HER2-amplified breast xenograft models and HPV + models [40, 122]. Preclinically, CDX-3379 in combination with cetuximab or BYL719 (a PI3Kα-selective inhibitor) enhanced growth inhibition in HNSCC xenograft models [124, 125]. In clinical trials, CDX-3379 alone or in combination with cetuximab was well tolerated and caused tumor regression in HNSCC [90, 91]. Other clinical trials have confirmed the safety profile of CDX-3379 combined with other HER therapies or vemurafenib [89, 92].

##### AV-203 (CAN017)

AV-203 is a humanized IgG1 mAb against HER3 that inhibits NRG binding [126–128]. AV-203 inhibits both ligand-dependent and ligand-independent HER3 signaling and downregulates HER3. This mAb has been reported to inhibit tumour growth in xenograft models derived from human NSCLC, breast, pancreatic, kidney, head and neck and esophageal cancer models. In a phase I clinical trial AV-203 demonstrated to be safe in metastatic or advanced solid tumors [93].

##### GSK2849330

GSK2849330 is a chimeric IgG1/IgG3, glycoengineered humanized mAb against subdomain III of HER3 [129]. Due to these modifications, this antibody has high binding affinity to FcγRIIIa and to human complement protein C1q, leading to enhanced ADCC and complement dependent cytotoxicity (CDC). This mAb blocks NRG binding and therefore receptor dimerization and activation. In vivo, GSK2849330 significantly reduces tumour growth in several xenograft models, including models with *NRG* alterations (fusion or overexpression) [94, 129, 130]. At present, it has been tested in two phase I clinical trials. In NCT01966445, GSK2849330 achieved a durable response in a unique responder with an oncogenic driver *CD74-NRG1-*rearranged molecular alteration present in a NSCLC tumor [94, 131].

##### Seribantumab (MM-121, SAR256212)

Seribantumab is a human IgG2 mAb that competes with NRG for binding to HER3. It blocks dimerization and induces HER3 internalization and degradation. MM-121 decreases tumour growth in pancreatic, ovarian (including cisplatin resistant models), prostate, kidney and *NRG1-*rearranged cancer models [71, 132–136]. In addition, multiple combinations of MM-121 with other anti-HER therapies have been analysed. The combination of MM-121 and trastuzumab inhibited cell growth in HER2 + breast cancer, including trastuzumab resistant models [137]. MM-121 also enhanced the antitumoral activity of chemotherapy in HER2 + breast cancer models resistant to paclitaxel and trastuzumab [138], and in cisplatin resistant ovarian cancer xenografts [135]. The combination of MM-121 plus erlotinib inhibited the proliferation of pancreatic cancer cells [132]. MM-121 in combination with letrozole resensitized to the latter drug in ER + breast cancer xenografts [139]. Finally, the combination of MM-121 and cetuximab inhibited growth in HNSCC models, including cetuximab resistant models [140, 141] and in engineered mouse models of lung cancers driven by EGFR T790M-L858R [134]. Seribantumab was generally well tolerated and combined safely with several drugs, but did not produce clinical benefit [97–100, 102, 103].

##### Patritumab (AMG-888, U3-1287)

Patritumab is a fully human IgG1 mAb that inhibits ligand binding to HER3 and induces receptor internalization and degradation [142]. Patritumab, alone or in combination with an anti-EGFR mAb, reduced NSCLC xenografts growth, including an EGFR TKI-resistant model [142, 143]. In addition, the combination of patritumab plus erlotinib overcame erlotinib resistance induced by NRG in NSCLC models [144]. Patritumab has also shown its potential as single agent and in combination with panitumumab in HNSCC cells and xenografts [145]. The combination of patritumab and radiation treatment enhanced radiation sensitivity in HNSCC and NSCLC [146]. This antibody was also effective against cetuximab resistance mediated by NRG in colorectal cancer [147]. In addition, patritumab in combination with trastuzumab and lapatinib potentiated tumor growth inhibition in HER2 + breast cancer models, including models resistant to trastuzumab [148]. Patritumab has shown capability to potentiate the antitumor activity of vincristine and cyclophosphamide in ES-4 Ewing’s sarcoma xenografts [149]. This mAb is currently being tested in phase I-III clinical trials with encouraging results [106, 107, 109, 150].

##### Elgemtumab (LJM716)

Elgemtumab or LJM716 is a fully human IgG1 mAb that binds to an epitope located between domains II and IV of the ECD of HER3, blocking the receptor in a closed conformation and preventing its activation [151]. This antibody inhibits tumor growth in both NRG-expressing and HER2 + cancer models, being more efficient in combination with other anti-HER therapies, such as cetuximab and trastuzumab. The combination of elgemtumab with trastuzumab and lapatinib significantly improved survival of mice with HER2 + breast cancer xenografts. When elgemtumab was given in combination with BYL719/alpelisib (PI3K inhibitor), they synergistically inhibited growth in HER2 + models, including trastuzumab-resistant HER2 + /*PIK3CA* mutant MDA-MB-453 xenografts [152]. In patients, LJM716 in combination with alpelisib and trastuzumab had antitumor activity but gastrointestinal toxicity [153]. However, this antibody demonstrated clinical activity and safety [110–113].

##### REGN1400

REGN1400 is a fully human IgG mAb that inhibits NRG binding and growth of epidermoid carcinoma, breast cancer and HNSCC cell lines and xenografts. REGN1400 in combination with anti-EGFR or anti-HER2 antibodies inhibits tumor growth more potently [154, 155]. REGN1400 in combination with erlotinib or cetuximab has been tested in a phase I trial and was well tolerated [114].

##### Sym013

Sym013 (Pan-HER) is a mixture of 6 mAbs, comprising 3 pairs of mAbs, each targeting EGFR, HER2 and HER3 [156]. This mixture promotes degradation of receptors, induces ADCC and CDC, has effect in the presence of ligands and inhibits activation of the PI3K and ERK pathways. Sym013 was tested in vivo and in vitro against an extensive panel of more than 100 cancer cell lines and in most cases was effective [156]. It is worth mentioning that Sym013 effectively inhibited growth of models resistant to chemotherapy and HER-targeted therapies (e.g., cetuximab, trastuzumab and T-DM1) [156–160]. The combination of Sym013 with single or fractionated radiation in NSCLC and HNSCC xenografts, including cetuximab resistant models, showed a potent antitumor effect and delayed regrowth [158]. Sym013 was under clinical development, but the clinical trial was terminated due to the inadequate safety profile [161].

#### In preclinical phase

A3 and A4 are humanized IgG1 mAbs targeting two different HER3 epitopes. These antibodies inhibit NRG binding, phosphorylation of HER3 and promote HER3 downregulation blocking its recycling [162, 163]. A3 and A4 are active in melanoma and pancreatic models, interfering with cell proliferation and migration [164, 165]. In addition, the combination of A3 and A4 with BRAF/MEK inhibitors potently inhibited cell growth and tumor relapse in a xenograft model [165]. Furthermore, combination of A3 with EGFR TKIs synergistically affected cell proliferation and inhibited tumor growth in lung cancer xenografts, including gefitinib-resistant models [166]. In addition, A3 has shown synergistic antitumor effect in combination with an HDAC inhibitor in NSCLC primary tumor cultures [167].

The anti-HER3 mouse mAb MP-RM-1 and its humanized version EV20 inhibit ligand-dependent and independent activation of HER3, promote its degradation, and inhibit HER2-HER3 dimerization. They have potent anti-tumor effects in breast, pancreatic, ovarian, melanoma and prostate cancer models [168, 169].

SGP1 is a mAb against HER3 and competes with NRG for binding HER3 [170]. This antibody reduces cell growth stimulated by NRG and increases growth inhibition in combination with trastuzumab in breast cancer cells [171]. SGP1, alone or combined with lapatinib, inhibited proliferation in parental and lapatinib-resistant HER2 + breast cancer cells [172].

The mouse monoclonal 9F7-F11 (non-ligand competitive) and the fully human IgG1 H4B-121 (NRG-competitive) antibodies recognize domain I and III of HER3 respectively, blocked HER2-HER3 dimerization and promote HER3 downregulation [173–175]. These antibodies, alone or in combination with anti-HER2 therapies, reduced tumor growth in epidermoid, pancreatic, lung, triple-negative breast cancer (TNBC) and HER2-low cancer cell xenografts.

Okita et al. have recently generated several anti-HER3 rat mAbs (Ab1-Ab7) which induce strong internalization of HER3, inhibition of NRG binding, HER3 phosphorylation and cell growth in several cancer cell lines. Ab4 shows effect in combination with erlotinib in HER2 + breast cancer and colorectal xenografts [176].

Anti-HER3^ECD^ [177] antagonizes NRG binding to HER3, increases its internalization, prevents HER2-HER3 dimerization and therefore cell proliferation and migration in invasive breast cancer cell lines [178]. Yosef Yarden’ lab generated mouse mAbs against the ECD of HER3 [179] that accelerate HER3 degradation and inhibit growth in vitro and in tumor-bearing animals, specially NG33 alone or in combination with other anti-HER3 Abs. This antibody is active in erlotinib-resistant models and prevents osimertinib resistance when given in combination with osimertinib and cetuximab in lung cancer models [180]. A mixture of three antibodies (called 3xmAbs) against EGFR, HER2 and HER3 was reported to be effective in lung cancer models resistant to second- and third-generation EGFR inhibitors, expressing mutant forms of EGFR. The triple mAbs combination triggered the degradation of receptors, inhibited cell proliferation, reduced tumor growth and sensitized these resistant cells to cisplatin and other TKIs. Combining 3xmAbs with a low dose osimertinib improved anti-tumor efficacy [181, 182].

1A5 antibody prevents ligand-independent activation of HER3 by binding to the HER3-ECD and 3D4 prevents ligand-dependent activation by blocking NRG binding. Both antibodies have modest antiproliferative activity but act synergistically with trastuzumab in HER2 + gastric models [183]. LMAb3 is an anti-HER3 mAb IgG1 that inhibits growth in an acquired trastuzumab-resistant ovarian cancer model [184].

Turowec et al. produced IgG 95, a synthetic antibody against open form of HER3 that blocks ligand binding and promotes HER3 ubiquitination, internalization, and downregulation. This antibody has anti-proliferative activity in HER2-amplified breast cancer cells and inhibits tumor growth in pancreatic xenografts [185].

Three mouse antibodies against HER3, HER3-3, HER3-8 and HER3-10, have been reported to be extremely potent in inhibiting basal proliferation and ligand-induced growth in breast cancer cell lines. HER3-8 and HER3-10 antibodies inhibited HER2-HER3 dimerization. For this reason, HER3-8 was selected to be humanized, and was termed huHER3-8 [186]. HuHER3-8 in combination with a BRAF inhibitor reduced tumor growth and increased durable response in mutant *BRAF* models of melanoma [187]. In addition, huHER3-8 reduced growth and signaling in wild-type BRAF/NRAS cutaneous melanomas [188].

IgG 3–43 is a HER3-targeting human antibody that recognizes an epitope between subdomains III and IV of HER3. It competes with NRG for binding to HER3, efficiently inhibits ligand dependent and independent HER3 activation and induces receptor internalization and degradation. IgG 3–43 showed efficacy in gastric, colorectal, lung, breast and HNSCC models [189, 190].

H3Mab‑17 is an IgG2a, kappa mAb generated by immunizing mice with HER3‑overexpressing cells. This antibody has ADCC and CDC properties and decreases growth in colon cancer models [191].

Hassani et al. generated several mouse mAbs against different HER3 extracellular subdomains with anti-proliferative effect on HER3-expressing cancer cells and some of them with synergistic effects in combination with trastuzumab [192].

Eliseev et al. developed single-domain antibodies that target the ECD of HER3 obtained originally from immunized llamas and which present anti-proliferative properties [193].

#### Limitations of mAbs targeting HER3

Although most of the mAbs have reported moderate clinical activity with toxicity manageable, clinical development for most of them has been discontinued. On the one hand, none of them reported clinically meaningful benefit. On the other hand, combination strategies have been limited either by toxicity [84], or by lack of efficacy [83, 97–100, 102, 105]. Bispecific antibodies (bAbs) and ADCs are expected to improve the clinical efficacy of anti-HER3 therapies.

### Bispecific antibodies

Bispecific antibodies target two different protein epitopes, either on the same protein or in different proteins. The latter may result in increased specificity of the antibody if the two epitopes are located on different proteins expressed or overexpressed in the tumoral tissue. In addition, if the antigen is located on immune cells, the bAb can facilitate the infiltration of immune system cells in the tumor. Table 2 summarizes clinical trials of bAbs against HER3.

**Table 2:** Bispecific antibodies against HER3 under clinical development

Study population	Clinical Trial, phase	Adverse events	Status, conclusion (references)
**Zenocutuzumab (Zeno, MCLA-128)**			
Solid tumors harboring an NRG1 fusion	NCT02912949, phase I + II	Infusion related reactions, diarrhea, rash and fatigue	Recruiting As of January 2017, MCLA-128 reported a safety profile and antitumor activity in pretreated metastatic BC patients progressing on HER2 therapies [194]
A patient with advanced NRG1-fusion positive solid tumor	NCT04100694, NA		Available
Metastatic BC	NCT03321981, phase II	Neutropenia/neutrophil count decrease, diarrhea, asthenia/fatigue and nausea	Active, not recruiting The combination of MCLA-128 + trastuzumab + vinorelbine is active in pretreated patients with HER2 + metastatic BC. The treatment is safe with manageable AEs mainly related to chemotherapy [195]
**SI-B001**			
Locally advanced or metastatic epithelial tumors	NCT04603287, phase I		Active, not recruiting
Recurrent and metastatic HNSCC	NCT05054439, phase II		Recruiting
Recurrent metastatic ESCC	NCT05022654, phase II		Recruiting
Recurrent and metastatic NSCLC	NCT05020769, phase II + III		Recruiting
EGFR/ALK wild-type recurrent or metastatic NSCLC	NCT05020457, phase II		Recruiting
Recurrent and metastatic HNSCC	NCT05044897, phase II		Recruiting
Unresectable or metastatic digestive system malignancies (colorectal and gastric cancer)	NCT05039944, phase II		Recruiting
**MM-111**			
Advanced, refractory HER2 amplified, NRG + BC	NCT01097460, phase I	Fatigue, diarrhea and dyspnoea	Completed. Study results available online
Advanced, refractory HER2 amplified, NRG + cancers	NCT00911898, phase I		Completed. Study results available online
Advanced HER2 + solid tumors	NCT01304784, phase I	Anemia, acute renal failure (assessed as cisplatin-related), chest pain, decreased appetite, diarrhea, febrile neutropenia, hyperuricemia, hypokalemia, hyponatremia, hypophosphatemia, mucosal inflammation, nausea, neutropenia, stomatitis, thrombocytopenia and vomiting	Completed. No results posted Treatment with MM-111 and standard of care HER2-directed regimens was viable [196]
HER2 + carcinomas of the distal esophagus, gastroesophageal junction and stomach	NCT01774851, phase II	Diarrhea, anemia, decreased appetite, alopecia, fatigue, nausea, vomiting, asthenia, neutropenia, constipation and cough	Terminated (DSMB recommendation due to lack of efficacy. There were no safety signals). Study results available online MM-111 did not improve PFS or OS when added to paclitaxel + trastuzumab [197]
**Istiratumab (MM-141)**			
Advanced solid tumors	NCT01733004, phase I	Vomiting, nausea, fatigue, abdominal pain, increased AP, dyspnea, diarrhea, anemia, increased AST and rash	Completed. No results posted MM-141 was well tolerated as monotherapy and in combination with everolimus or paclitaxel + gemcitabine in patients with relapsed/refractory solid tumors [198]
Metastatic pancreatic cancer	NCT02399137, phase II	Neutropenia, alopecia, diarrhea, fatigue, thrombocytopenia, anemia and decreased appetite	Completed. No results posted Istiratumab failed to improve the efficacy of chemotherapy [199]
CRC, NSCLC and HNSCC	NCT02538627, phase I		Terminated (Sponsor decision). No results posted
**Duligotuzumab (MEHD7945A, RG7597)**			
Locally advanced or metastatic solid tumors with mutant KRAS	NCT01986166, phase I	Diarrhea, general disorders, dermatitis acneiform, rash, rash erythematous, rash maculo-papular and nausea	Completed. No results posted The combination of cobimetinib and duligotuzumab was related with increased toxicity and limited efficacy [200]
Locally advanced or metastatic epithelial tumors	NCT01207323, phase I	Headache, rash and diarrhea	Completed. No results posted Duligotuzumab was well-tolerated with evidence of tumor pharmacodynamic modulation and anti-tumor activity in HNSCC [201]
Recurrent/metastatic HNSCC	NCT01911598, phase I	Neutropenia, hypokalemia, dehydration, anemia and diarrhea in arm A and neutropenia, anemia, febrile neutropenia, leukopenia, thrombocytopenia and hypomagnesemia in arm B	Completed. No results posted Duligotuzumab with cisplatin + 5-fluorouracil (arm A) or carboplatin + paclitaxel (arm B) demonstrated promising activity despite chemotherapy dose reductions and could be maintained with duligotuzumab alone [202]
KRAS wild-type metastatic CRC	NCT01652482, phase II	Rash, diarrhea, fatigue and nausea. There were fewer rash events of any grade in the duligotuzumab arm but more diarrhea	Completed. No results posted The combination of FOLFIRI with duligotuzumab generally did no improve clinical outcomes benefit compared with cetuximab combination [203]
Recurrent/metastatic HNSCC	NCT01577173, phase II	Rash, infections, diarrhea, fatigue and nausea	Completed. No results posted Duligotuzumab demonstrated similar activity to cetuximab, but not superior [204]

#### Under clinical development

##### Zenocutuzumab (Zeno, MCLA-128)

Zenocutuzumab is a bAb IgG1 targeting HER2 (domain I) and HER3 (domain III) [205]. Zenocutuzumab has a ‘dock and block’ mechanism: docks to HER2 and blocks ligand binding to HER3 and therefore inhibits oncogenic signaling via HER2-HER3 heterodimers. The mechanism of action of this bAb includes enhanced ADCC activity due to the glycoengineered modification of the IgG1. This bAb has shown efficacy in breast, gastric and pancreatic cancer models, including models resistant to HER2-directed therapies (trastuzumab and T-DM1) and in the presence of high concentrations of NRG. Zenocutuzumab inhibited growth of *NRG1* fusion-positive cancer models, also demonstrating efficacy in patients with chemotherapy-resistant *NRG1* fusion-positive metastatic cancer [206]. Zenocutuzumab is currently being tested in phase I/II clinical trials, which reported well tolerated safety profile as well as anti-tumor activity [194, 195].

##### SI-B001

SI-B001 is an IgG-(scFv)2 bAb that targets EGFR and HER3. This bispecific tetravalent antibody is based in the model of an IgG-(scFv)2 structure that consists of a complete IgG with two heavy and two light chains, and two scFv components connected to either C or N terminals of the heavy or light chains [207]. SI-B001 has recently demonstrated its efficacy in colon, HNSCC and esophageal cancer xenograft models, achieving almost complete inhibition of the growth in the last two models [208]. SI-B001 is now being tested in phase I and II clinical trials.

##### MM-111

MM-111 is a bAb directed to HER2 and HER3 in which the anti-HER2 arm localizes the bAb in HER2 + tumor cells and the anti-HER3 arm blocks NRG binding [209, 210]. This bAb is synthesized as single polypeptide fusion protein of two human scFv binding arms, targeting HER2 and HER3, linked to modified human serum albumin. In preclinical studies, this bAb decreased growth in HER2 + gastric, breast, ovarian, and lung cancer models and demonstrated an increased antitumor activity combined with trastuzumab or lapatinib in HER2 + breast cancer. In a clinical trial, this bAb reported to be safe also in combination with standard of care HER2-targeting drugs and chemotherapy [196]. However, the phase II clinical trial NCT01774851 in HER2 expressing gastroesophageal cancers was terminated early due to lack of effect of MM-111 plus paclitaxel and trastuzumab [197]. Because of this disappointing result, all further studies investigating MM-111 were revoked.

##### Istiratumab (MM-141)

Istiratumab is a tetravalent bAb holding 4 high-affinity binding sites, two are specific for IGF1R and two for HER3 [211–213]. Structurally, istiratumab contains an IgG1 mAb against IGF1R that was engineered to contain two single-chain Fv fragments targeting HER3 fused at the C terminus of the heavy chain. Notably, istiratumab blocks ligand binding (NRG and IGF-1/2), downregulates receptor levels and suppresses downstream signaling. Istiratumab demonstrated its potential inhibition of growth in multiple models including pancreatic, sarcoma, renal, ovarian, melanoma and prostate cancer. This bAb potentiated the anti-tumoral effects of chemotherapy and of the mTOR inhibitor everolimus in models of pancreatic and ovarian cancer [211, 212, 214]. Istiratumab has been evaluated in clinical trials with disappointing results [199].

##### Duligotuzumab (MEHD7945A, RG7597)

Duligotuzumab is a humanized bAb IgG1 targeting EGFR and HER3 that blocks ligand binding, inhibits signaling pathways and potentiates ADCC [215, 216]. Duligotuzumab contains two identical Fabs that can bind EGFR or HER3. Duligotuzumab strongly inhibited tumor growth in several preclinical models, including human epidermoid carcinoma, pancreatic, breast, colorectal, HNSCC and lung cancer, especially in combination with chemotherapy. Duligotuzumab demonstrated its efficacy in resistant models to erlotinib and cetuximab derived from HNSCC and NSCLC in monotherapy [217] or in combination with cisplatin [218]. Its action has also been reported in combination with AKT and PI3K inhibitors in TNBC [219]. In addition, duligotuzumab enhanced the antitumor effect of trastuzumab in HER2 + gastric models [220]. Recently, it has been reported that duligotuzumab increased ionizing radiation response in cervical cancer models [221]. Several clinical trials (phases I/II) are testing duligotuzumab and in general reported limited activity [200–204].

#### In preclinical phase

Tab6 or TA is a tetravalent and bAb against HER2 and HER3 that consists in the anti-HER2 antibody trastuzumab fused with HER3-specific scFvs derived from a seribantumab biosimilar called Ab6 in its both CH3 domains [222]. Surprisingly, treatment with TAb6 increased the proliferation of HER2 + breast cancer cell lines. However, in the presence of NRG, TAb6 in combination with lapatinib significantly reduced proliferation. In addition, Tab6 restored sensitivity to the PI3K inhibitor GDC-0941 in prostate cancer cells resistant to that inhibitor [223].

A5/F4 is an oligoclonal mixture of two IgGs based on scFv against domains I (F4) and III (A5) of HER3. A5/F4 inhibits ligand-dependent HER3 signaling, cell proliferation and enhances the activity of HER-targeted agents in vitro and in vivo [224].

Bispecific molecules called dual variable domain immunoglobulin (DVD-Ig) proteins against EGFR and HER3 have also been developed [225]. These molecules consist of a human IgG1 heavy chain and Igκ light chain constant domains linked with an additional variable domain (VH and VL sequences) at the N terminus of both Fab arms. The HER3-targeting variable domains of the DVD-Igs are derived from seribantumab. In vitro, anti-EGFR/HER3 DVD-Ig proteins were superior inhibiting growth in comparison to parental mAbs combination or a conventional bAb.

Recently, Rau et al. have generated a tetravalent and bAb called scDb hu225 × 3–43-Fc targeting both EGFR and HER3 [226]. This antibody is composed of a bispecific single-chain diabody (scDb) generated by the antigen-binding site of the humanized version of cetuximab (IgG hu225) and the IgG 3–43 (described above) fused to the hinge region of a human Fcγ1 chain (scDb-Fc). Its efficacy blocking proliferation, inhibiting HER phosphorylation, downstream signaling and inducing receptor internalization and degradation has been demonstrated in HNSCC and TNBC models. Indeed, this bAb in combination with trastuzumab is also effective in colorectal cancer models, bypassing NRG-induced resistance to anti-EGFR therapies [227]. The same lab had also generated Dab-Fc 2 × 3 molecule, an innovative bivalent and bispecific molecule (Dab-Fc) that targets HER2 and HER3 with anti-tumoral activity in vitro and in vivo [228]. Dab-Fc comprises the variable domains of trastuzumab (anti-HER2 Ab) and IgG 3–43 (anti-HER3 Ab) assembled into a diabody-like construction stabilized by CH1 and CL domains and fused to a human γ1 Fc region. Recently, IgG 3–43 was used to generate novel and effective scDb‑based trivalent bispecific antibodies directed against HER3 and CD3 that target T‑cells to HER3-expressing cancer cells [229, 230].

1G5D2 is a native bispecific hybridoma mAb with dual specificity for HER3 and HER2 ECDs that strongly inhibited cell proliferation alone or in combination with trastuzumab [231].

### Antibody–drug conjugates

ADCs are a new class of antitumoral agents designed to merge the selectivity of mAbs with the cell killing properties of a cytotoxic drug (payload) attached by a linker to the mAb. That linker may be cleavable or non-cleavable [232, 233].

#### Under clinical development

##### U3-1402 (Patritumab deruxtecan, HER3-DXd)

U3-1402 also called patritumab deruxtecan or HER3-DXd is an ADC composed by patritumab covalently conjugated to a drug-linker containing deruxtecan, a topoisomerase I inhibitor [234]. U3-1402 was efficiently internalized, induced HER3 degradation and showed growth inhibition activity in HER3 + breast, gastric and colorectal cancer [234, 235]. U3-1402 is also effective alone or in combination with an EGFR-TKI in EGFR-TKI-resistant NSCLC models, in which EGFR inhibition with osimertinib pretreatment increased U3-1402 efficacy [236–238]. Recently, it has been demonstrated that U3-1402 sensitized HER3 + tumors to programmed cell death-1 (PD-1) blockade [239]. Patritumab deruxtecan has demonstrated its clinical efficacy in metastatic *EGFR*-mutated NSCLC, after disease progression on EGFR TKI therapy [240]. U3-1402 is currently under clinical evaluation (Table 3) and has demonstrated antitumor activity and manageable safety profile in breast cancer and *EGFR*-mutant NSCLC [240–245].

**Table 3:** Antibody Drug-Conjugates against HER3 under clinical development

Study population	Clinical Trial, phase	Adverse events	Status, conclusion (references)
**U3-1402 (Patritumab deruxtecan, HER3-DXd)**			
Advanced or metastatic CRC	NCT04479436, phase II		Terminated (Study was terminated early given the Interim Analysis for Part 1 (signal finding) did not meet pre-specified criteria and will not proceed to Part 2. Sponsor will proceed closing the study). No results posted
Naïve patients with HR + /HER2- early BC	NCT04610528, phase I		Recruiting
Metastatic or unresectable NSCLC	NCT03260491, phase I	Nausea, vomiting, fatigue, decreased appetite and alopecia	Recruiting U3-1402 has antitumor activity and manageable safety profile [240, 241, 244]
HER3 + metastatic BC	NCT02980341, phase I + II	Nausea, vomiting and decreased appetite	Active, not recruiting In a preliminary analysis, U3-1402 demonstrated antitumor activity and manageable safety profile [242, 245]
Metastatic or locally advanced EGFR-mutated NSCLC	NCT04619004, phase II		Recruiting
Locally advanced or metastatic EGFR-mutated NSCLC	NCT04676477, phase I		Recruiting
Metastatic BC	NCT04699630, phase II		Recruiting
Advanced BC	NCT04965766, phase II		Recruiting
Metastatic or locally advanced EGFR-mutated NSCLC after failure of EGFR TKI therapy	NCT05338970, phase III		Recruiting

#### In preclinical phase

Gianluca Sala’s group has generated several ADC versions derived from the anti-HER3 antibody EV20: (1) EV20-Sap obtained by coupling the plant toxin saporin, (2) EV20/MMAF, and (3) EV20‑sss‑vc/MMAF, by coupling the cytotoxic drug monomethyl auristatin F with non-cleavable or cleavable linker respectively and (4) EV20/NMS-P945 by coupling EV20 with a DNA minor groove alkylating agent (thienoindole NMS-P528) through a cleavable linker. EV20-Sap has cytotoxic activity in melanoma cells and reduces pulmonary metastases in a murine metastatic model of melanoma [246]. EV20/MMAF demonstrated HER3-dependent cell killing activity in melanoma and in HER2 + breast cancer cell lines and xenografts, including several models of cells resistant to anti-HER2 therapies [247, 248]. EV20/MMAF in combination with PLX4720 in *BRAF* mutated melanoma, and EV20/MMAF alone or plus vemurafenib resulted in an effective anti-metastatic activity in vivo. EV20‑sss‑vc/MMAF demonstrated its efficacy in HER3 + liver cancer [249]. Recently, EV20/NMS-P945 showed cytotoxic activity on prostate, HNSCC, pancreatic, melanoma, gastric and ovarian cancer [250].

The anti-HER3 antibody 9F7–F11 had been conjugated with monomethyl auristatin E to generate a novel ADC, MMAE–9F7–F11. This ADC increased arrest in G_2_/M, which is the most radiosensitive phase of the cell cycle and promoted cell death of HER3 + pancreatic cancer cells [251]. In vivo, MMAE–9F7–F11 in combination with radiation therapy increased the overall survival in a pancreatic cancer mouse model.

### Antibody-Derived molecules in preclinical phase

Hu et al. developed tetraspecific antibodies called FL518 and CRTB6 that recognize EGFR, HER2, HER3 and VEGF [252]. CRTB6 was generated by combining the variable regions of cetuximab, trastuzumab, lumretuzumab and bevacizumab into a DVD-Ig–like antibody and FL518 by combining the two bispecific antibodies duligotuzumab (against HER3 and EGFR) and bH1-44 (against HER2 and VEGF). These tetraspecific antibodies were more effective inhibiting signaling and growth than bispecific antibodies in colorectal, breast, pancreatic, lung or gastric cancer models, including anti-HER-resistant cancer cells.

TsAb2v2 and TsAb3v1 are tetraspecific, tetravalent Fc-containing antibodies targeting EGFR, HER3, cMet and IGF1R generated by the combination of N-terminal single-chain Fabs and C-terminal single-chain Fvs in an IgG1 antibody format [253]. The binding arms are derived from imgatuzumab (EGFR), lumretuzumab (HER3), onartuzumab (cMet) and R1507 (IGF1R). These antibodies bind and inhibit all targets at the same time and show higher apoptosis induction and tumor growth inhibition over mAbs or bAbs in pancreatic, breast and lung tumor models.

Trispecific ErbB-cMet-IGF1R antibodies which target EGFR, IGF1R and cMet or EGFR, IGF1R and HER3 have been reported to inhibit receptor activation and cellular growth [254].

Alternative anti-HER3 antibody-derived formats that provide a similar binding capacity but with improved properties, such as a small size and higher tissue penetration and extravasation have been developed [255]. Among these several novel molecules derived from antibody structures are surrobodies. They are comprised of a diversified immunoglobulin heavy chain and an invariant surrogate light chain that together confer specific high-affinity binding to their targets. Two of these surrobodies, SL-175 and SL-176, reduced growth of several tumor models in vitro and in vivo, and were even more potent in combination with trastuzumab and lapatinib in HER2 + cell lines [256]. Affibodies are three-helix bundle Z-domain based on such domain of staphylococcal protein A that have short plasma half-life time and rapid clearance with low production cost [257]. Recently, several anti-HER3 affibody molecules have been reported with activity in pancreatic and ovarian cancer models [258, 259]. ICG-Z_Her3_ is a dimeric HER3-specific affibody coupled to a photosensitizer (indocyanine green) that mediated photothermal therapy (transform light into heat energy to kill cancer cells) and had antitumoral properties in HER3 + cancers [260]. Bispecific affibodies against HER3 and HER2 in which two affibodies were linked by an albumin‐binding domain have also been generated [261]. A novel platform developed diabody-Ig and generated active tetravalent bAbs against EGFR and HER3 [262]. The antigen-binding site of these molecules is composed of a diabody in the VH-VL orientation stabilized by fusion to antibody-derived homo- or heterodimerization domains, further fused to an Fc region.

### Pan-HER tyrosine kinase inhibitors (pan-TKIs) under clinical development

Due to the reported low activity of the HER3 kinase domain and the requirement of heterodimerization with other HER receptors for its activation, blocking the receptor partners leads to the suppression of HER3 activity. This means that pan-TKIs, which inhibit catalytic activity of HER members, indirectly act as HER3 inhibitors as well [263, 264]. In this review we will not focus on this family of agents.

### Other anti-HER3 strategies for cancer therapy

#### Under clinical development

##### HER3 vaccine

At present, there are two clinical trials using HER3 vaccines. NCT03832855 is a phase I clinical trial that uses an investigational cancer vaccine called pING-hHER3FL. pING-hHER3FL is a circular piece of DNA that produces the full length human HER3 protein. On the other hand, NCT04348747 is a phase II trial study that uses a dendritic cell vaccine against HER2-HER3, in combination with other drugs that may boost the immune system to recognize and destroy cancer cells.

#### In preclinical phase

In preclinical studies, a vaccine generated with an adenovirus encoding the full length human HER3 receptor (Ad-HER3 or Ad-HER3-FL) has been evaluated preclinically [265, 266]. Ad-HER3 induced strong T-cell anti-tumor responses and anti-HER3 antibodies that have effectiveness against breast cancer, including models of acquired resistance to HER2-targeted therapies. High efficacy of Ad-HER3-FL in combination with dual PD-1/PD-L1 and CTLA4 blockade treatments has also been reported.

Miller et al. evaluated four HER3 peptides of the HER3-ECD as putative B-cell epitopes to activate the immune system and produce highly specific HER3 antibodies [267]. They reported enhanced anti-tumor effects of these HER3 vaccine antibodies in breast and pancreatic cancer preclinical models. They also reported enhanced response and higher levels of ADCC when the HER3 vaccine antibodies are combined with HER2, HER1 and IGF1R vaccine antibodies.

RB200 is a bispecific ligand trap which binds to HER3 ligand NRG and EGFR ligands [268]. This molecule was generated combining the EGFR and HER3 ligand binding domains with an Fc fragment of human IgG1. RB200 prevents ligand-dependent receptor activation and inhibits proliferation in vitro and in xenograft models.

Several antisense oligonucleotides or microRNAs have been described to be able to downregulate HER3 and inhibit proliferation. EZN-3920 is a HER3 antisense oligonucleotide which has anti-tumor activity alone or combined with TKIs in vitro and in xenograft tumor models, including models of resistance to anti-HER therapies [269]. Several miRNAs such miR-125a, miR-125b, miR-205 and miR-450b-3p suppress HER3 expression by directly targeting 3′ UTR of HER3 mRNA and inhibit proliferation of breast cancer cells [270–272].

HER3 siRNAs decrease cell proliferation and sensitize cells to anti-HER therapies [79, 273]. In addition, several authors had developed carriers to direct siRNAs or drugs to cancer cells. For example, HER3 aptamers, artificial single-stranded DNA or RNA oligonucleotides that bind HER3, have been used to target HER3 + tumoral cells. Yu et al. reported the antitumoral action of a three-in-one nucleic acid aptamer-siRNA chimera that targets EGFR-HER2-HER3 in HER2 + breast cancer [274]. Recently, Shu et al. demonstrated the antiproliferative activity of carbon dots/HER3 siRNA, alone or in combination with trastuzumab in HER2 + breast cancer cells [275]. HER3 aptamer-protamine-siRNA (against oncogenes or CDKs) nanoparticles have anticancer effect in HER3 + breast cancer models [276]. In addition, a HER3 aptamer-functionalized liposome encapsulating doxorubicin has been developed to deliver it in HER3 + models [277]. Sorafenib encapsulated in microparticles with anti-HER3 aptamers in the surface diminish the toxicity of sorafenib [278]. An RNA aptamer against HER3-ECD, A30, inhibited NRG signaling and therefore cell growth in breast cancer cells [279]. A30 was also used to deliver a set of cytotoxic siRNAs and inhibit growth in HER3 + breast cancer cells [280]. Recently, a novel RNA aptamer called HBR has been reported to inhibit HER3/NRG interaction [281].

Xie et al. reported ATP-competitive small molecule inhibitors targeting the pseudokinase of HER3 that can perturb the biological function of HER3 [282, 283]. TX1-85–1 interacts with Cys721 in the ATP-binding pocket of HER3 but has a poor effect in proliferation and HER3-dependent functions in vitro. However, a derivate of TXI-85–1 with a hydrophobic adamantane moiety, TX-121–1, produces covalent modification of HER3, causes partial degradation of HER3, interferes the dimerization of HER3 with c-Met and HER2 and perturbs HER3-dependent signaling and growth.

Sims et al. synthesized a polypeptide called HerPBK10 or HPK which had a minimal receptor binding domain constructed from the structure of NRG1 [284]. It specifically binds to HER3. HPK is inert and it was used to deliver a variety of therapeutic payloads, generating HPK-nanobiologics that mimic the natural ligand-receptor interaction on HER3 but resulting in delivery of a tumor-toxic molecule. For instance, they used doxorubicin to generate H3-D and a sulfonated corrole generating H3-G. These HPK-nanobiologics are effective against trastuzumab-resistant models in a HER3-dependent manner.

Targeting HER3 ligand NRG could be an approach to block this receptor. For example, 7E3 is an antibody directed to NRG1 IgG-like domain that blocks NRG1-dependent growth in pancreatic cancer models [285]. This antibody decreases ligand-induced activation and expression level of HER3 and induces ADCC. There are other anti-NRG antibodies in preclinical stage, such as YW538.24.71 and YW526.90.28 [286].

## Conclusions

In this review it has been summarized several therapies against HER3, most of them in preclinical development. However, nowadays no treatment specifically targeting HER3 has been approved for clinical use. The therapeutic efficacy of an anti-HER3 regimen could be enhance by its combination with other anti-HER therapy, chemo-, immuno-, or radio-therapy. This fact has also been observed with anti-HER2 therapies, because for optimal inhibition of HER2 function in HER2 + breast cancer cells, treatment with at least two anti-HER2 drugs is required. It is hoped that anti-HER3 ADC approach would overcome the shortcomings of mAb-based HER3 therapy, with potent delivery of therapeutics payload to HER3 expressing cancer cells. Indeed, the generation of molecules derived from antibodies with low production cost, short plasma half-life time and rapid clearance have emerged in the field. However, the development of potent prognostic and predictive biomarkers for anti-HER3 targeted therapeutics is also required.

## Data Availability

Not applicable.

## Abbreviations

ADC: Antibody–drug conjugate
ADCC: Antibody-mediated cell-dependent cytotoxicity
AE(s): Adverse event(s)
bAbs: Bispecific antibodies
BC: Breast cancer
CDC: Complement dependent cytotoxicity
DLT: Dose-limiting toxicity
DSMB: Data and Safety Monitoring Board
DVD-Ig: Dual variable domain immunoglobulin
ECD: Extracellular domain
EGFR: Epidermal growth factor receptor
ER +: Estrogen receptor positive
ESCC: Esophageal squamous cell carcinoma
HER2 +: HER2-amplified
HNSCC: Head and neck squamous cell carcinoma
HPV +: Human papillomavirus positive
HRG: Heregulin
ID_50 _and ID_90_: The 50% and 90% inhibitory mass doses
IGF1R: Insulin-like growth factor 1 receptor
IMA: Invasive mucinous adenocarcinomas
ITT: intention-to-treat
mAbs: Monoclonals antibodies
NRG(s): Neuregulin(s)
NSCLC: Non-small cell lung cancers
OS: Overall survival
Pan-TKIs: Pan-tyrosine kinase inhibitors
PD-1: Programmed cell death-1
PFS: Progression-free survival
PR: Partial response
RAI: Radioactive iodine
RP2D: Recommended phase 2 dose
RTK: Receptor tyrosine kinases
scDb: Bispecific single-chain diabody
T-DM1: Trastuzumab-emtansine
TKIs: Tyrosine kinase inhibitors
TNBC: Triple-negative breast cancer
